# Controlling Antimicrobial Resistance through Targeted, Vaccine-Induced Replacement of Strains

**DOI:** 10.1371/journal.pone.0050688

**Published:** 2012-12-05

**Authors:** Yonas I. Tekle, Kaare M. Nielsen, Jingzhou Liu, Melinda M. Pettigrew, Lauren A. Meyers, Alison P. Galvani, Jeffrey P. Townsend

**Affiliations:** 1 Department of Epidemiology and Public Health, Yale University School of Medicine, New Haven, Connecticut, United States of America; 2 Department of Ecology and Evolutionary Biology, Yale University School of Medicine, New Haven, Connecticut, United States of America; 3 Department of Pharmacy, University of Tromsø, Tromsø, Norway; 4 Genøk-Center for Biosafety, Research Park, Tromsø, Norway; 5 Graduate Program in Microbiology, Yale University, New Haven, Connecticut, United States of America; 6 Section of Integrative Biology, The University of Texas at Austin, Austin, Texas, United States of America; 7 Santa Fe Institute, Santa Fe, New Mexico, United States of America; 8 Biology Department, Spelman College, Atlanta, Georgia, United States of America; Fudan University, China

## Abstract

Vaccination has proven effective in controlling many infectious diseases. However, differential effectiveness with regard to pathogen genotype is a frequent reason for failures in vaccine development. Often, insufficient immune response is induced to prevent infection by the diversity of existing serotypes present in pathogenic populations of bacteria. These vaccines that target a too narrow spectrum of serotypes do not offer sufficient prevention of infections, and can also lead to undesirable strain replacements. Here, we examine a novel idea to specifically exploit the narrow spectrum coverage of some vaccines to combat specific, emerging multi- and pan-resistant strains of pathogens. Application of a narrow-spectrum vaccine could serve to prevent infections by some strains that are hard to treat, rather than offer the vaccinated individual protection against infections by the pathogenic species as such. We suggest that vaccines targeted to resistant serotypes have the potential to become important public health tools, and would represent a new approach toward reducing the burden of particular multi-resistant strains occurring in hospitals. Vaccines targeting drug-resistant serotypes would also be the first clinical intervention with the potential to drive the evolution of pathogenic populations toward drug-sensitivity. We illustrate the feasibility of this approach by modeling a hypothetical vaccine that targets a subset of methicillin-resistant *Staphylococcus aureus* (MRSA) genotypes, in combination with drug treatment targeted at drug-sensitive genotypes. We find that a combined intervention strategy can limit nosocomial outbreaks, even when vaccine efficacy is imperfect. The broader utility of vaccine-based resistance control strategies should be further explored taking into account population structure, and the resistance and transmission patterns of the pathogen considered.

## Introduction

The evolution of antimicrobial drug resistance is a major impediment to infectious disease control. Disease prevention methods such as improved hygiene and vaccine developments have proven successful in controlling many diseases. However, the effectiveness of vaccines depends on the standing and evolving variability of serotypes in a given population of bacterial pathogens. Future disease prevention that relies on vaccine development is frequently limited by inability to induce adequate immune responses to the full range of disease-causing serotypes of a particular pathogenic species present in a given environment. For instance, the design of a single vaccine that produce immune responses against *Streptococcus pneumonia* has proven challenging given the presence of more than 90 disease-causing serogroups [1], [2], [3]. An alternative approach to seeking broad coverage is to develop vaccines with a narrower spectrum, specifically targeting only the most problematic pathogenic strains. However, effectiveness in the short run may in the long run be by-passed by negative effects due to strain replacement; that is, an increased prevalence of pathogenic strains of the same species that are not controlled by the available vaccine. Several empirical studies and mathematical models have examined the concerns and consequences of vaccine-induced pathogen strain replacement [4], [5], [6], [7], [8], [9], [10].

We propose to exploit strain replacement, until now viewed as a negative effect of vaccination, to specifically control multi-and pan-resistant serotypes of pathogens. Here we explore the feasibility of applying a new narrow spectrum vaccine that primarily serves to prevent infections by specific strains that are hard to treat, rather than to offer the vaccinated individual protection against all infections by the pathogen species. Rather than offering broad protection, currently the aim of most vaccine development, the proposed narrow-spectrum vaccines would offer protection from infection with particular resistant serotypes. Such vaccines would serve important public health goals, and offer a much-needed new tool for reducing the morbidity and mortality of certain nosocomial infections. Targeting drug-resistant serotypes is also the first clinical intervention that could act to drive the evolution of pathogenic populations toward drug-sensitivity. We illustrate the feasibility of this approach by modeling a hypothetical vaccine that targets a subset of methicillin-resistant *Staphylococcus aureus* (MRSA) serotypes, in combination with drug treatment targeted at drug-sensitive serotypes.

MRSA causes life-threatening infections such as pneumonia, meningitis, endocarditis, and septicemia [11], [12]. Recently, MRSA has undergone extensive epidemiological expansion in hospitals [13] and in communities [14]. The resulting mortality has even surpassed that from HIV infection in the US [15]. Since its emergence in the early 1960’s, the prevalence of MRSA and the spectrum of its resistance to antimicrobials have dramatically increased [16]. Few effective antibiotics to treat MRSA infections remain. Moreover, the speed at which this pathogen is evolving resistance indicates that current treatment options could soon prove futile. The creation of a single vaccine that effectively protects against the diversity of MRSA populations is unlikely to be practical [17], [18], [19], [20], [21]. The projected lack of treatment and vaccine options exemplifies the urgent need to develop alternative and complementary approaches to infection control. Here we explore the option of developing a targeted narrow spectrum vaccination, in this case targeted toward a subset of evolutionarily related MRSA strains, could be one new approach. Our case study also serves to identify the many variables related to the population structure, and the resistance and transmission patterns of the species that are required for modeling of resistance-targeting vaccines. The future practical utility of vaccine-based resistance control strategies may well be for other bacterial species than the one considered in our case study.

Two types of epidemiologically important MRSA isolates are generally recognized: community-associated MRSA (CA-MRSA) and health care-associated MRSA (HA-MRSA) [22], [23], [24], [25]. However, the evolutionary history of the ∼359 MRSA genotypes that have been identified is complex [16]. Genome-level analysis is providing an understanding of MRSA relationships and facilitating the identification of the origin and emergence of epidemiologically important and expanding clones [16], [26]. It is now possible to identify clonal complexes of epidemiologically important and emerging populations of multi-drug resistant MRSA sequence types that can be targeted for narrow-spectrum vaccination. A narrow-spectrum vaccine has the potential to be targeted toward specific multi-drug resistant MRSA clonal complexes and serotypes that either demonstrate a higher proclivity for multi-drug resistance, or that have already been established as a key cause of morbidity and mortality in particular regions [see [27].

To assess the effect of combining hospital-based vaccination against a multi-drug resistant genotype with treatment for drug-sensitive genotypes, we developed a transmission model of two MRSA genotypes: a multi-drug resistant genotype against which a vaccine is targeted (the Vaccine-Targeted Genotype, VTG) and a genotype that exhibits less drug resistance against which treatment is still effective (the Treatment-Targeted Genotype, TTG). We evaluated a control strategy based on VTG vaccination of hospital patients and the treatment of TTG. We determined the impact of this combined intervention strategy on nosocomial outbreaks of the two MRSA genotypes. We find in our case study that a combined intervention strategy can control outbreaks of drug-resistant strains in hospitals. The vaccine-induced shift in the selection pressure in favor of less drug-resistant genotypes, also illustrated the prospects for long-term control of resistant clones.

## Methods

### The Model

We modeled the transmission dynamics of two MRSA genotypes (VTG and TTG) within the hospital. Patients were separated into compartments corresponding to susceptible (*S*, including in treatment, *S_t_*, or vaccinated, *S_v_*), colonized (*C*, including less resistant TTG-MRSA, *C_l_*, or more resistant VTG-MRSA, *C_h_*), and infected (*I*, including TTG-MRSA, *I_l_*, or VTG-MRSA, *I_h_*, Eqs 1–10, Fig. S1). All newly admitted patients (Λ, susceptible, λ*_cl_*, colonized with TTG, λ*_ch_*, colonized with VTG), with the exception of infected patients (λ*_il_*, infected with TTG, λ*_ih_*, infected with TTG), are vaccinated (*θ*, *θ_h_* for VTG, and *θ_l_* cross-immunity for TTG) thus entering into the vaccinated compartmental structures (Eqs 1–5, Fig. S1A), while incoming infected patients and already admitted patients that did not receive vaccination enter the unvaccinated compartments (Eqs 6–10, Fig. S1B). Vaccination coverage (*χ*) for the newly admitted patients is also accounted for in the model. Hospitalized patients exit (*η*) from any compartments via discharge or death. Infected patients are treated with effective antimicrobial drugs (*μ*). Susceptible patients can become colonized from both colonized (*β_cl_* or *β_ch_*) and infected (*β_il_* or *β_ih_*) individuals from the vaccinated and unvaccinated groups (Eqs 1, 6), and can progress to infection (*φ*) with the respective pathogens (Eqs 4, 5, 9, 10). For simplicity, co-infection by both genotypes is not considered, with the observation that competitive exclusion by the dominant genotype often occurs [28], [29]. Colonization by two or more strains has been reported [30] and mathematically analyzed in relation to competitive exclusion [31]. Here we focus on infection rates for which no evidence exists for simultaneous co-infections from multiple strains [32].

Steady state model equations for vaccinated (*v*) group:
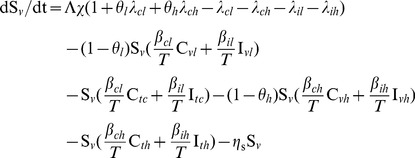
(1)

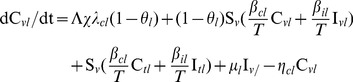
(2)

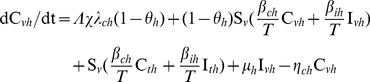
(3)

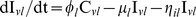
(4)

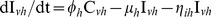
(5)


Steady state model equations for unvaccinated (*t*) group:
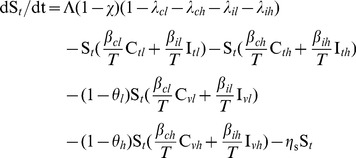
(6)

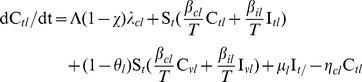
(7)

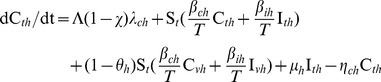
(8)

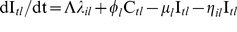
(9)

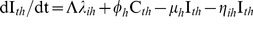
(10)


### Analysis

Parameter values used in our model for both genotypes of CA-MRSA (here treated as TTG) and HA-MRSA (here treated as VTG) were obtained from published data estimated from Beth Israel Deaconess Medical Center tertiary hospital and the literature [33]. Baseline estimates take into account the biology of both MRSA genotypes for transmissibility and conditions of patients’ health (susceptible, colonized and immunocompromised) for length of stay in the hospital. Detailed account and mathematical analyses of baseline values used in the study are given in D’Agata et al. [33] and Webb et al. [28], respectively. To accommodate different estimates in the literature, we explored a wide range parameter values, assessing the impact of our chosen intervention measures on controlling MRSA outbreaks. Moreover, we emphasize the main purpose of the presented study and chosen model is to illustrate the feasibility of the narrow-target vaccine to a relevant case-example. We used the same assumptions as in Webb et al. [28], [33] and calculated steady states with and without admittance of colonized (*λ_c_*) and/or infected (*λ_i_*) patients. Basic reproductive numbers for the low resistant genotype TTG (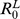) and highly resistant genotype VTG (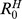) were numerically computed to determine possible outbreaks due to one or both genotypes (see Appendix S1). We also examined the long-term behavior of the model by varying related parameter values concurrently. Baseline values are tabulated in Table S1 and the detailed series of differential equations used to calculate steady states are provided in Equations 1–10.

Our hypothetical vaccine is assumed to confer some or no cross-immunity against the less resistant genotypes, TTGs. Vaccination is administered for each patient as they are admitted into the hospital. We evaluated vaccine efficacy, cross-immunity, and coverage against both MRSA genotypes. Vaccination is given at admission for all susceptible and colonized patients. Infection arising before the vaccine takes an effect is accounted for by the efficacy (Appendix S1). We analyzed the steady state to evaluate the long-term effect of vaccine in controlling outbreaks in hospitals. The baseline transmissibility values were chosen to show the maximal effect of individual and combined intervention measures in controlling over all MRSA outbreaks (Table S1). Moreover, we also considered a range of transmissibility values for more realistic application of the model to observed MRSA dynamics in the hospital (see [33].).

Treatment intervention strategy with effective antimicrobial agents against TTG is used for patients infected with these genotypes. We analyzed the impact of vaccination and treatment across a range of efficacy values on the transmission dynamics of MRSA genotypes.

## Results

### Vaccination Against VTG-MRSA

**Figure 1:**
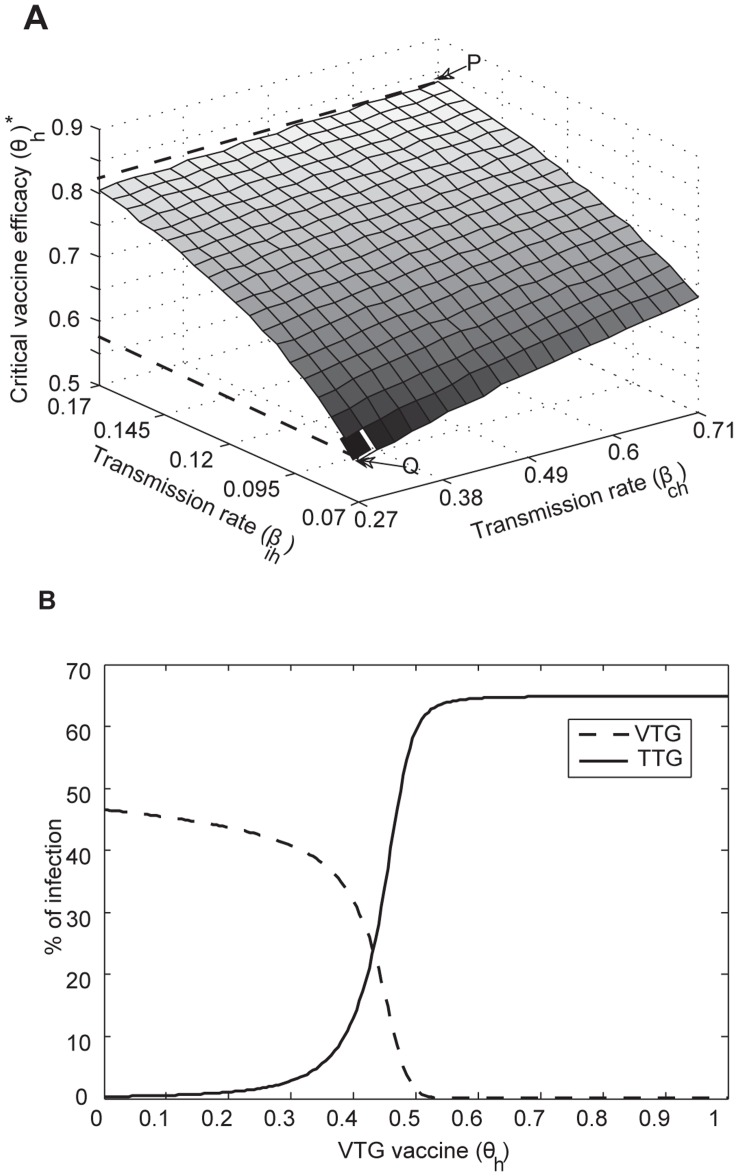
The effect of vaccination on the dynamics of VTG-MRSA in absence of treatments against TTG-MRSA. (A) Critical vaccination efficacy values (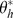) required to eliminate VTG-MRSA with varying values of transmission rates (*β_ch_* and *β_ih_*). Points P and Q show the critical values required to stop VTG outbreaks in highest and lowest transmission values, respectively. (B) The effect of vaccine and competition, when VTG has higher *R*0 (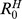 *= *5.91) compared to TTG (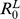 *= *4.11). As the efficacy of vaccine increases VTG is replaced by TTG.

Vaccination can effectively eliminate VTG over a range of vaccine efficacies (Fig. 1A; Table S1). The critical levels of vaccine efficacy required to control a VTG-MRSA outbreak without other interventions, including no treatment and cross-immunity against TTG, depend on the transmissibility of VTG (Fig. 1). For example, when VTG transmissibility is high (*β_ih = _*0.17 and *β_ch_* = 0. 71), a vaccine efficacy of 83% is required to eliminate VTG (Fig. 1A). When VTG transmissibility is low (*β_ih = _*0.27 and *β_ch_* = 0.07), a vaccine efficacy of 56% is required to eliminate VTG (Fig. 1A).

We analyzed the impact of TTG co-circulation and vaccination when VTG is more transmissible than TTG 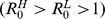 (Fig. 1B). In this setting VTG infection declines with increased vaccination efficacy and steeply decreases as TTG increases. The vaccination efficacy required to eliminate VTG (50%) is reduced by the concomitant competition of TTG (Fig. 1B).

### Treating TTG-MRSA

**Figure 2:**
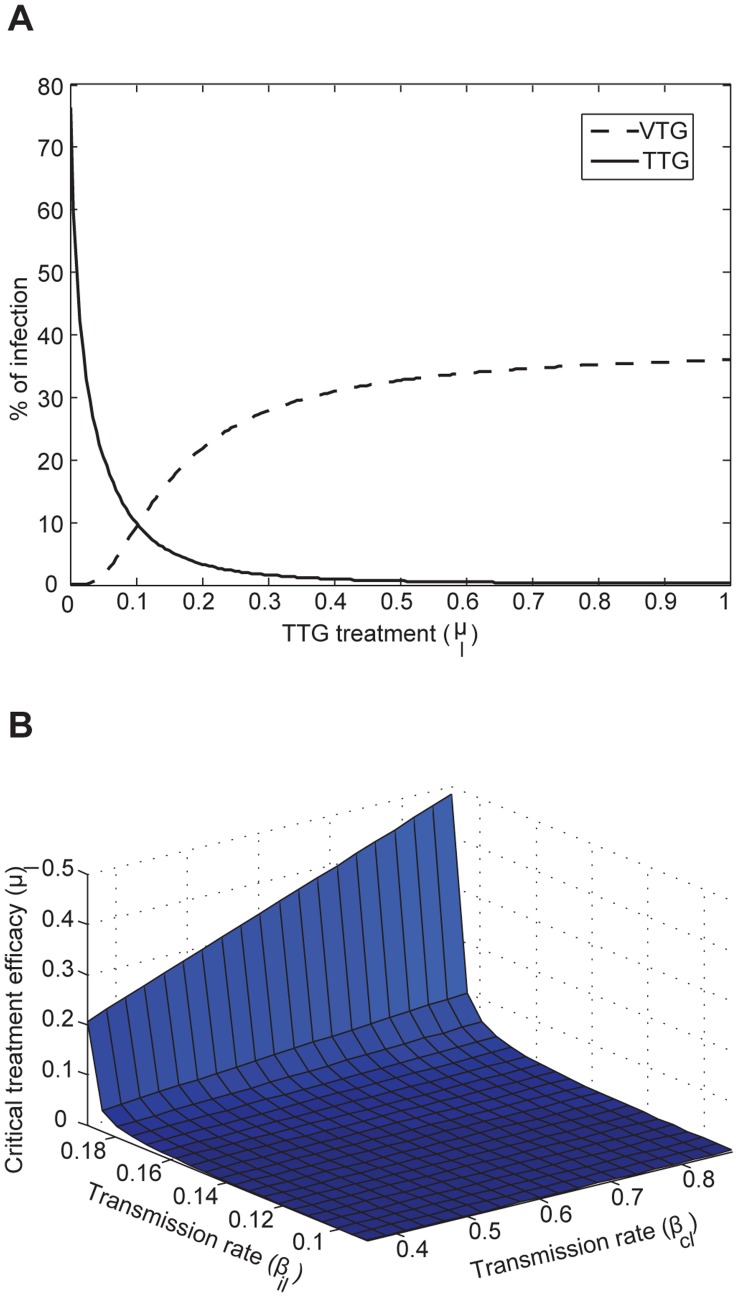
Treatment and vaccine cross-immunity targeted at TTG-MRSA. (A) Effect of different treatment (*µ_l_*) values on TTG, when the competing pathogen, VTG-MRSA, is left untreated. VTG (*I_h_*) infection increases as TTG infection (*I_l_*) is eliminated with increased efficacy of treatment. In this analysis baseline and moderate (*β_ih = _*0.55, *β_ch_* = 0.13) transmission rates for TTG and VTG are used, respectively. (B) Effects of treatment (*µ_l_*) and cross-immunity (*θ_l_ = *77.2%) at different TTG transmission rates (*β_il_* and *β_cl_*). Minimum treatment is required to stop TTG outbreak when a critical cross-immunity value is used (77.2%).

When treatment is effective against TTG and when VTG is resistant to treatment (Fig. 2), TTG infection will continually drop with increased efficacy of treatment, while VTG increases rapidly depending on its transmissibility. For moderate transmissibility of VTG (*β_ch = _*0.55, *β_ih_* = 0.13) and baseline transmissibility of TTG (*β_cl_ = *0.87, *β_il_* = 0.19), VTG infection increases from ∼0% to 36% (Fig. 2A). Although the initial prevalence of TTG, due to its faster generation time and larger reservoir in community [33], is higher than VTG, treatment tips the competitive advantage in favor of VTG, resulting in the elimination of TTG (Fig. 2A).

**Figure 3:**
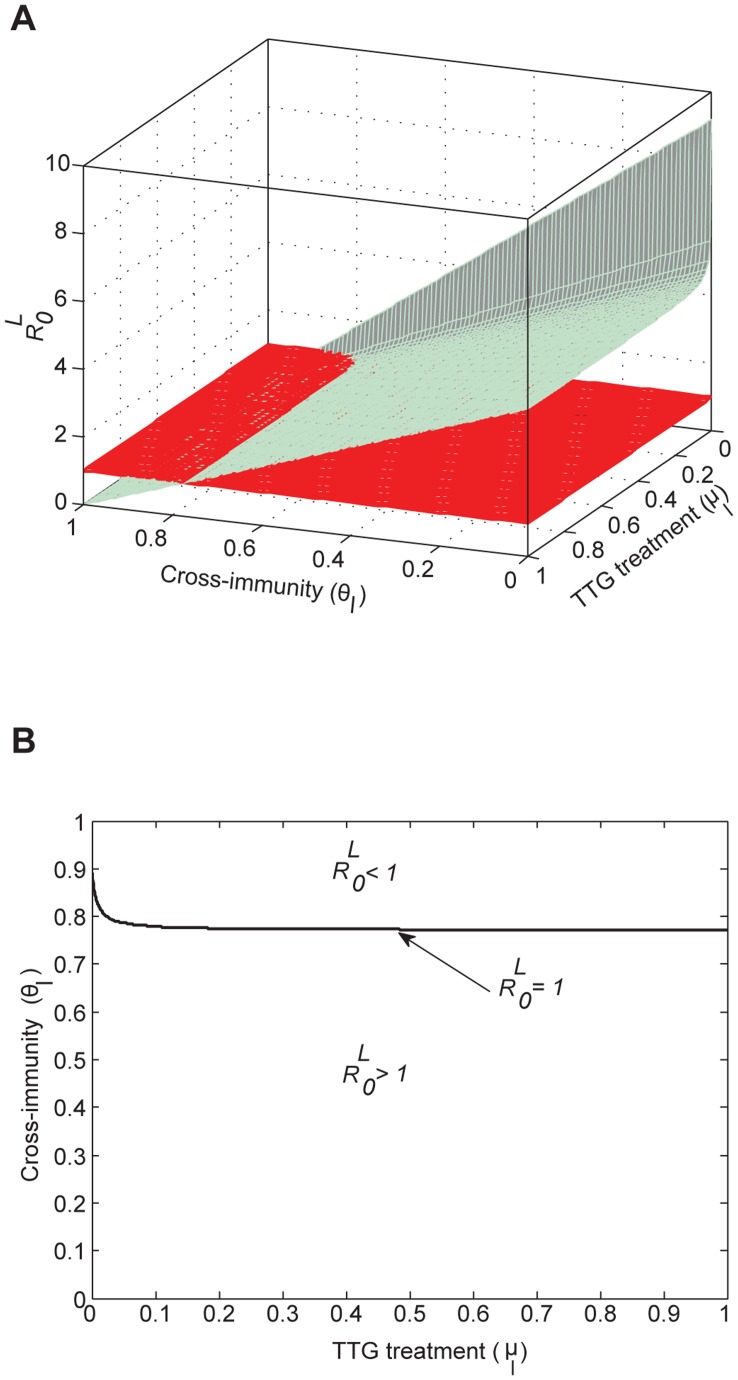
The effect of combined interventions, treatment and cross-immunity, in controlling outbreaks of TTG. (A) Different values of treatment (*µ_l_*) and vaccine cross-immunity (*θ_l_*) and basic reproductive number of TTG (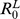). Note that the overall eradication of TTG outbreak 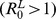 and progression of colonized individuals to infection is dependent on efficacy of cross-immunity (*θ_l_*). (B) Critical combined intervention values required to stop TTG outbreak. Minimal treatment efficacy (<30%) and 77% cross-immunity are required to stop TTG outbreak 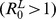.

**Figure 4:**
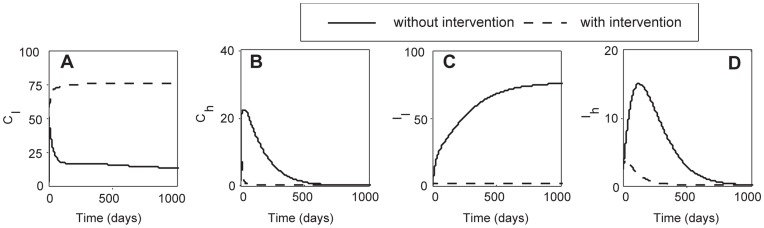
Baseline steady state analyses before and after combined intervention strategies (*µ_c = _*1 and *θ_h = _*1) (A–D). (A) TTG colonization (*C_l_*). The colonization rate of TTG is greatly reduced but not eliminated. Additional intervention, cross-immunity, is required to completely clear TTG colonization. (B) VTG colonization (*C_h_*). VTG colonization is eliminated faster after intervention. However, VTG colonization can be reduced in absence of intervention by competitive exclusion. (C). TTG infection (*I_l_*) is eliminated after combined intervention. (D) VTG infection (*I_h_*) is eliminated with intervention but can also go extinct through time as a result of competitive exclusion in absence of intervention. These analyses do not include admission of colonized or infected patients.

Treatment can effectively reduce TTG infection including at higher transmission rates (Fig. 3B). For example, when TTG transmissibility is high (*β_ih = _*0.19 and *β_ch_* = 0.87), less than 30% treatment efficacy is required to eliminate TTG (Fig. 1B). However, colonization persists even at higher (100%) levels of treatment efficacy (Fig. 4A) due to the ineffectiveness of treatment in clearing colonized patients. To completely eradicate TTG outbreak in the hospital 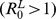, we require vaccine cross-immunity (∼77%) for a wide range of TTG transmission rates (Figs. 3A, B, S2).

### Combined Interventions: Treatment, Cross-immunity and Vaccine

For the baseline transmissibility (Table S1), both TTG and VTG have basic reproductive numbers greater than one 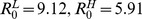; Fig. 4A–D). As a result of its higher transmissibility, TTG drives VTG close to extinction prior to intervention (Fig. 4B, D).

Maximal combined use of vaccination and treatment is predicted to eliminate TTG infection but not colonization (Fig. 4A), whereas such an intervention combination was found to easily eradicate VTG infection and colonization (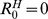; Fig. 4B, D). Our analysis shows that the complete elimination of the TTG outbreak hinges on reducing the number of colonized patients, which could be achieved if the vaccine elicits cross-immunity against TTG (Figs. 3A, B, S2C, D). For baseline parameters, treatment will eliminate TTG infection but not colonization (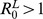; Fig. 3A). However, the use of treatment with some vaccine cross-immunity (≥72.2% efficacy) will eradicate the pathogen altogether 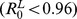 (Fig. 3A, B).

When we include admission of colonized or infected patients (Figs. S3, S4), we find a general increase (from 10% to 78%) of patients colonized with TTG following combined interventions (Fig. S2A, I). However, the rise in TTG colonization is reduced greatly by enhanced efficacy of vaccine cross-immunity against TTG (Figs. 3, S2A, 4A). The number of VTG-colonized patients generally decreases after combined interventions (Figs. 3B, S3B, J), but remains nearly constant when infected patients are admitted (Figs. S3F, S4F). This inflow of infected patients into the system maintains a constant level of VTG-colonized patients, whereas the percentage of VTG-infected patients increases (Figs. S3H, S4H). The increase in infection occurs due to the reduced competition from TTG as a result of treatment and the continued influx of infected patients. The prevalence of TTG dramatically decreases (from between 64–80% to 0–1%) under combined interventions (Figs. S3C, G, K, S4C, G, K).

## Discussion

We have developed and examined the outcomes of a mathematical model of the effects of a narrow spectrum vaccine that specifically target the most prevalent drug resistant genotypes. We show in our model that a hospital-based, targeted vaccination can have an immediate impact in terms of protecting exposed patients, as well as a long-term impact by counterbalancing positive selection of resistance in particular clones/strains.

The practical utility of vaccine-based resistance control strategies remains to be explored, and will depend on the population and resistance biology of the particular pathogen. However, examples from the scientific literature suggest that the approach may be considered for further development. Effective serogroup-specific vaccines have previously been designed for some bacterial pathogens including the seven-valent pneumococcal conjugate vaccine and the *Haemophilus influenzae* type B vaccine [34], [35]. Similar efforts could therefore be made to design and develop vaccines that target the most drug-resistant serotypes and other emerging pathogens causing severe burdens in hospitals including *Enterococcus faecium* [27]. Epidemiological and geographic [36] characteristics must be taken into account, possibly leading to different vaccine targets, target strains, designs and composition for different regions.

In our model, competitive release within the ecological niche is expected when one genotype is left untreated while the other is treated. The community associated MRSA (here denoted TTG) has a competitive advantage over the hospital genotype (here denoted VTG) due to a reservoir of TTG in the community and faster generation time [33], [37]. Competitive exclusion resulting in removal of one genotype might occur when the *R*_0_ of the dominating genotype is greater than one and that of the competing genotype is less than one. However, competitive exclusion alone is not sufficient to halt outbreaks due to either genotype. Recent mathematical modeling that incorporated co-colonization of multiple strains argued against a large effect of competitive exclusion, demonstrating that co-colonization could become endemic over time [31]. Accordingly, our analysis demonstrates that TTG colonization remains endemic at lower cross-immunity efficacy values - even when competitive exclusion is considered. Our analysis indicates that combined control strategies can effectively curtail MRSA outbreaks and suppress endemicity, even when there is a continual influx of colonized and/or infected patients (see Appendix S1).

Previous mathematical models of pneumococcal vaccination have indicated that using a genotype-specific vaccine might change the ecological niche and result in replacement of the invading genotype by the non-vaccine genotypes [38], [39]. Our model suggest even a vaccine with relatively low efficacy is able to reduce the transmissibility of the most drug-resistant MRSA population. Consequently, genotype-specific vaccines can be used to limit the prevalence and control the emergence of drug resistance. Thus, the objective, the degree of serotype coverage, and the efficacy of appropriate vaccines can differ fundamentally from the requirements of most other vaccine development pipelines that aim to achieve broad coverage of all relevant serotypes. Most currently available vaccines against bacterial agents have gained regulatory approval due to their efficient protection against disease rather than as being efficient tools to prevent infection by a narrow subset of pathogenic genotypes. Here we apply a mathematical model to understand the possible utility of a narrow-range targeted vaccine in terms of both short-term and long-term control of particular serotypes.

Our model suggests that vaccination prior to or upon hospital admission can be an effective approach to control hospital outbreaks. However, some properties of vaccine efficacy might influence the short-term outcome. Immunity often takes several days to build subsequent to vaccination. In our model, this immunity lag period following vaccination was included in the parameter for vaccine efficacy (Appendix S1). The efficacy of the vaccine, assuming full protection after 10 days, was 78% and 91% in high and low transmission rates, respectively (Appendix S1). Furthermore, actual efficacy could be higher than assumed in our calculations because many patients at risk of MRSA infection could be vaccinated (and re-vaccinated) prior to scheduled hospital admissions, thereby ensuring sufficient time to build immunity before exposure to nosocomial serotypes. Indeed, vaccinating patients prior to admission could be an effective strategy for controlling outbreaks and limit other costly prevention options such as screening, isolation and decolonization [40], [41], [42].

One limitation of the proposed strategy would be the extent that a particular serotype is linked to resistance determinants; however, we maintain that many of the troublesome clinical strains/clones do also present particular immunologically relevant cell surface properties due to common ancestry and/or as a consequence of synergistic survival, virulence, and resistance properties [21]. A recent study identified a potential evolutionarily conserved target and demonstrated its potential application for passive immunization of orthopedic MRSA infections [43]. Our study encourages further population genetic studies of microbial pathogens as well as further identification of surface properties that are associated with particular strains and their antibiotic-resistance traits.

Another potential issue with our approach is that incentives available for developing such narrow spectrum vaccines may not be equal to those offered by the market for broad infection control. However, many narrow-spectrum vaccines are produced as a consequence of broad-spectrum efforts; once developed, a smaller hospital-based market would still allow recouping of already-spent development costs. Moreover, public health policies may be put into place to incentivize the development of vaccines that present limited free market opportunities due to limited lifespan (resistant strain dynamics), geographical relevance, and size of targeted at-risk populations/groups.

The biology and transmission dynamics of MRSA are far more complex than most models can directly incorporate [28], [33], [44]. Intrinsic transmissibility values might be different from the baseline depending on the strains and on the inclusion of additional transmission routes from the environment, which could lead to an underestimate of the overall transmissibility. To accommodate these uncertainties, we considered wide ranges of transmissibility values that capture the potential long-term effects of intervention measures. We also ran simulations in which *R_0_* values were alternately higher for each of the genotypes. In all cases our results demonstrate that combined intervention is a powerful approach to the eradication of MRSA outbreaks including when transmissibility of either MRSA genotypes is high. Though multiple infections by more than one MRSA genotype have not been reported [32], some studies have indicated that co-colonization of more than one strain could occur simultaneously in an individual [30]. This co-colonization might correspond to weak levels of the competitive exclusion considered in our model [31]. However, our model focuses on the effects of intervention measures on pre- and post-colonization (infection) using vaccination and treatment, respectively. Strain replacement following intervention and due to competitive advantage is well established in many disease systems [2], [5], [6], [9], [45], [46], [47], [48]. A recent study also reports an absence of *Staphylococcus* polyclonal bacteremia in community sampled population indicating that competitive exclusion might play a role in MRSA infections [32]. Accordingly, the strain coverage of candidate vaccines is highly relevant to pragmatic implementation of our findings.

Targeting drug-resistant serotypes, as advocated here, would represent the first clinical intervention that could demonstrably act to drive the evolution of pathogenic populations toward drug-sensitivity. The generality of this approach as presented argues that it would also be applicable to other microorganisms (e.g. viruses and parasites), in any circumstance where vaccine-based approaches can target particularly undesirable phenotypes in the larger population of the pathogen, such as those that have developed reduced drug sensitivity.

## Supporting Information

Figure S1 **Schematic representation of the compartmental model, showing MRSA dynamics in unvaccinated groups (** ***A*** **) and vaccinated groups (** ***B*** **).** These two compartmental structures interact through transmission parameters. The total sum of the whole population in these structures is used to calculate the final steady state of each compartment. VTG: vaccine targeted multi-drug resistant MRSA genotypes, TTG: treatment targeted fewer (less) drug resistant MRSA genotypes. See for parameter symbols.
(PDF)

Figure S2 **Baseline steady state analyses before and after combined intervention strategies including cross-immunity (** ***µ_c =_*** **1,** ***θ_l_ =*** **77.2% and** ***θ_h =_*** **1) (** ***A–D*** **).** (*A*) TTG colonization (*C_l_*). (*B*) TTG infection (*I_l_*). (*C*) VTG colonization (*C_h_*). (*D*) VTG infection (*I_h_*). This analysis does not include admission of colonized or infected patients.
(PDF)

Figure S3 **Steady state analyses before and after combined intervention strategies (** ***µ_c_*** **= 1 and** ***θ_h_*** **= 1) at baseline with admission of colonized patients (** ***λ_l_*** **= 0.05,** ***λ_h_*** **= 0.007) (** ***A*** **–** ***D*** **), with admission of infected (** ***λ_il_*** **= 0.005,** ***λ_ih_*** **= 0.0017) (** ***E*** **–** ***H*** **) and with admissions both infected and colonized patients (** ***λ_cl_*** **= 0.03,** ***λ_ch_*** **= 0.07,** ***λ_il_*** **= 0.005,** ***λ_ih_*** **= 0.0017) (** ***I*** **–** ***L*** **).** See Table S1 for other baseline values.
(PDF)

Figure S4 **Steady state analyses before and after combined intervention strategies including cross-immunity (** ***µ_c =_*** **1,** ***θ_l_ =*** **77.2% and** ***θ_h =_*** **1) (at baseline with admission of colonized patients (** ***λ_l_*** **= 0.05,** ***λ_h_*** **= 0.007) (** ***A*** **–** ***D*** **), with admission of infected (** ***λ_il_*** **= 0.005,** ***λ_ih_*** **= 0.0017) (** ***E*** **–** ***H*** **) and with admissions both infected and colonized patients (** ***λ_cl_*** **= 0.03,** ***λ_ch_*** **= 0.07,** ***λ_il_*** **= 0.005,** ***λ_ih_*** **= 0.0017) (** ***I*** **–** ***L*** **).** See Table S1 for other baseline values.
(PDF)

Table S1 **Parameter values for the transmission dynamics of Methicillin-Resistant** ***Staphylococcus aureus*** **genotypes, obtained from D’Agata et al. (25), and additional parameters used in this study.**
(DOCX)

Appendix S1 **Steady State,** ***R_0_*** **and vaccine efficacy (EV) analyses.**
(PDF)
