# Supplementary material for: Controlling Antimicrobial Resistance through Targeted, Vaccine-Induced Replacement of Strains
Source: PLoS One. 2012 Dec 5;7(12):e50688. doi: 10.1371/journal.pone.0050688 (PMC3515573; doi:10.1371/journal.pone.0050688)
Supplement: Figure S1 — Schematic representation of the compartmental model, showing MRSA dynamics in unvaccinated groups ( A ) and vaccinated groups ( B ). These two compartmental structures interact through transmission parameters. The total sum of the whole population in these structures is used to calculate the final steady state of each compartment. VTG: vaccine targeted multi-drug resistant MRSA genotypes, TTG: treatment targeted fewer (less) drug resistant MRSA genotypes. See for parameter symbols. (PDF) [file pone.0050688.s001.pdf]

Figure S1

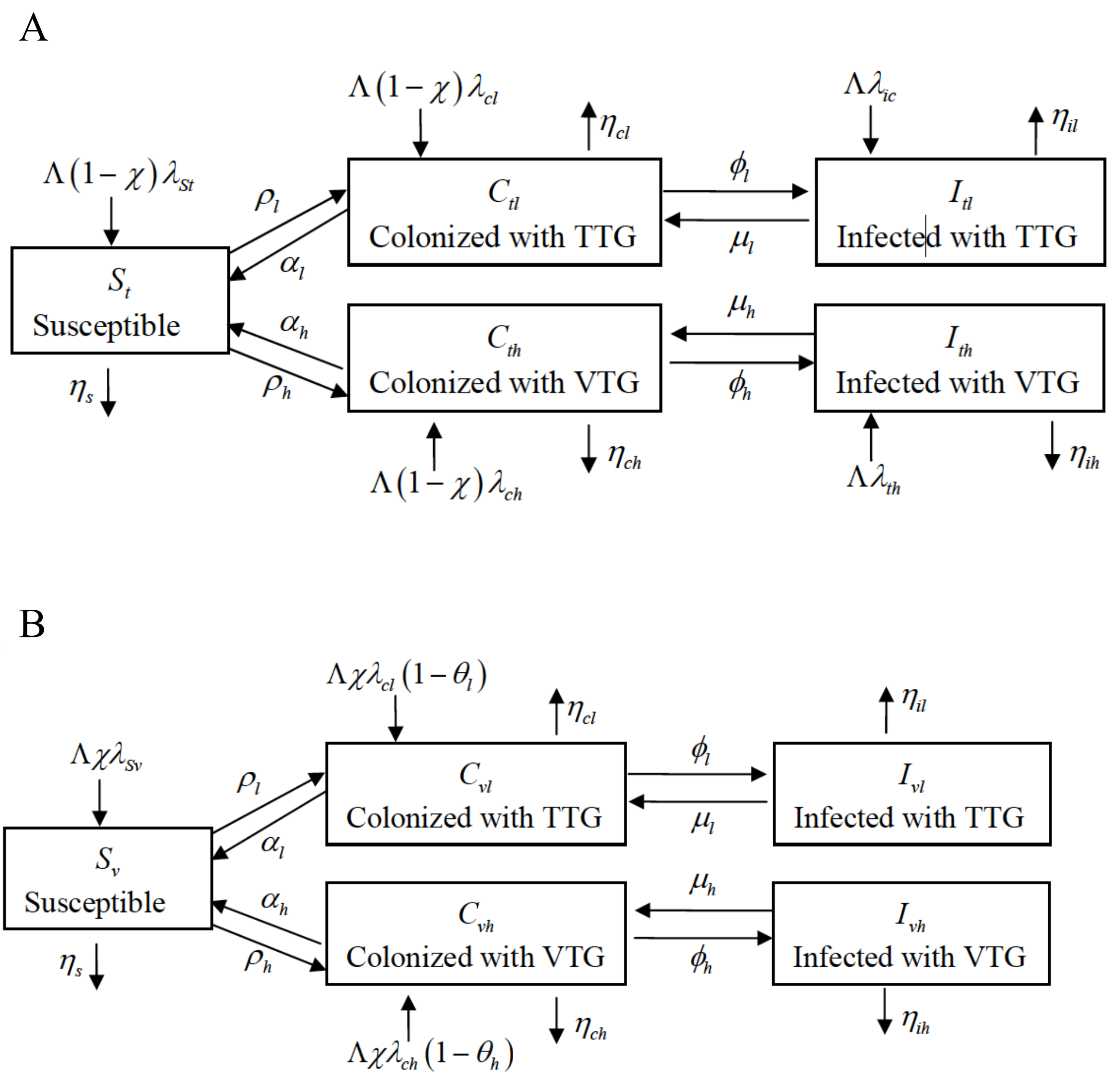

$\lambda_{St} \equiv 1-\lambda_{cl}-\lambda_{ch}-\lambda_{il}-\lambda_{ih}$ $\qquad$ $\lambda_{Sv} \equiv \lambda_{St}+\theta_l\lambda_{cl}+\theta_h\lambda_{ch}$

$\rho_l \equiv \frac{1}{T}\left[\left(\beta_{cl}C_{tl}+\beta_{il}I_{tl}\right)+\left(1-\theta_l\right)\left(\beta_{cl}C_{vl}+\beta_{il}I_{vl}\right)\right]$ $\qquad$ $\rho_h \equiv \frac{1}{T}\left[\left(\beta_{ch}C_{th}+\beta_{ih}I_{th}\right)+\left(1-\theta_h\right)\left(\beta_{ch}C_{vh}+\beta_{ih}I_{vh}\right)\right]$
