# Supplementary figures and images for: Controlling Antimicrobial Resistance through Targeted, Vaccine-Induced Replacement of Strains
Source: PLoS One. 2012 Dec 5;7(12):e50688. doi: 10.1371/journal.pone.0050688 (PMC3515573; doi:10.1371/journal.pone.0050688)

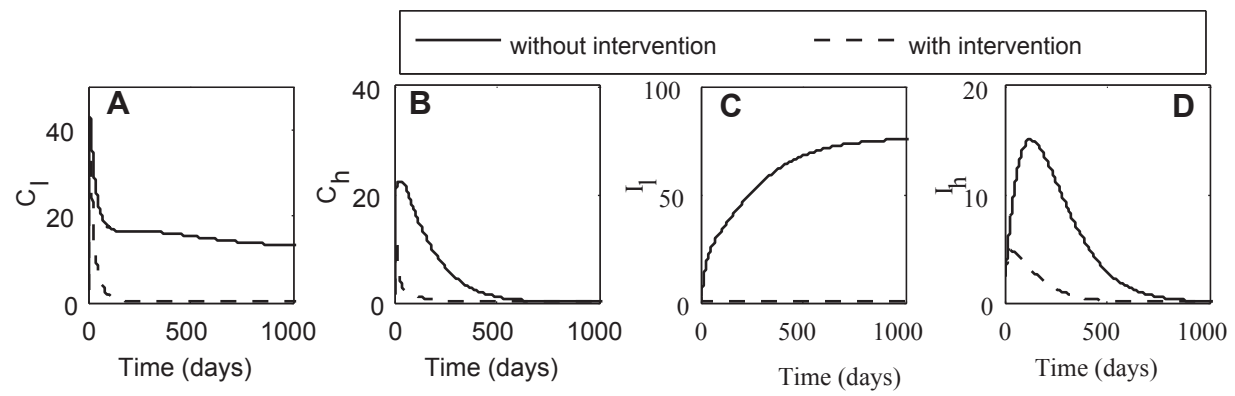

Supplement: Figure S2 — Baseline steady state analyses before and after combined intervention strategies including cross-immunity ( µc = 1, θl = 77.2% and θh = 1) ( A–D ). (A) TTG colonization (Cl). (B) TTG infection (Il). (C) VTG colonization (Ch). (D) VTG infection (Ih). This analysis does not include admission of colonized or infected patients. (PDF) [file pone.0050688.s002.pdf]

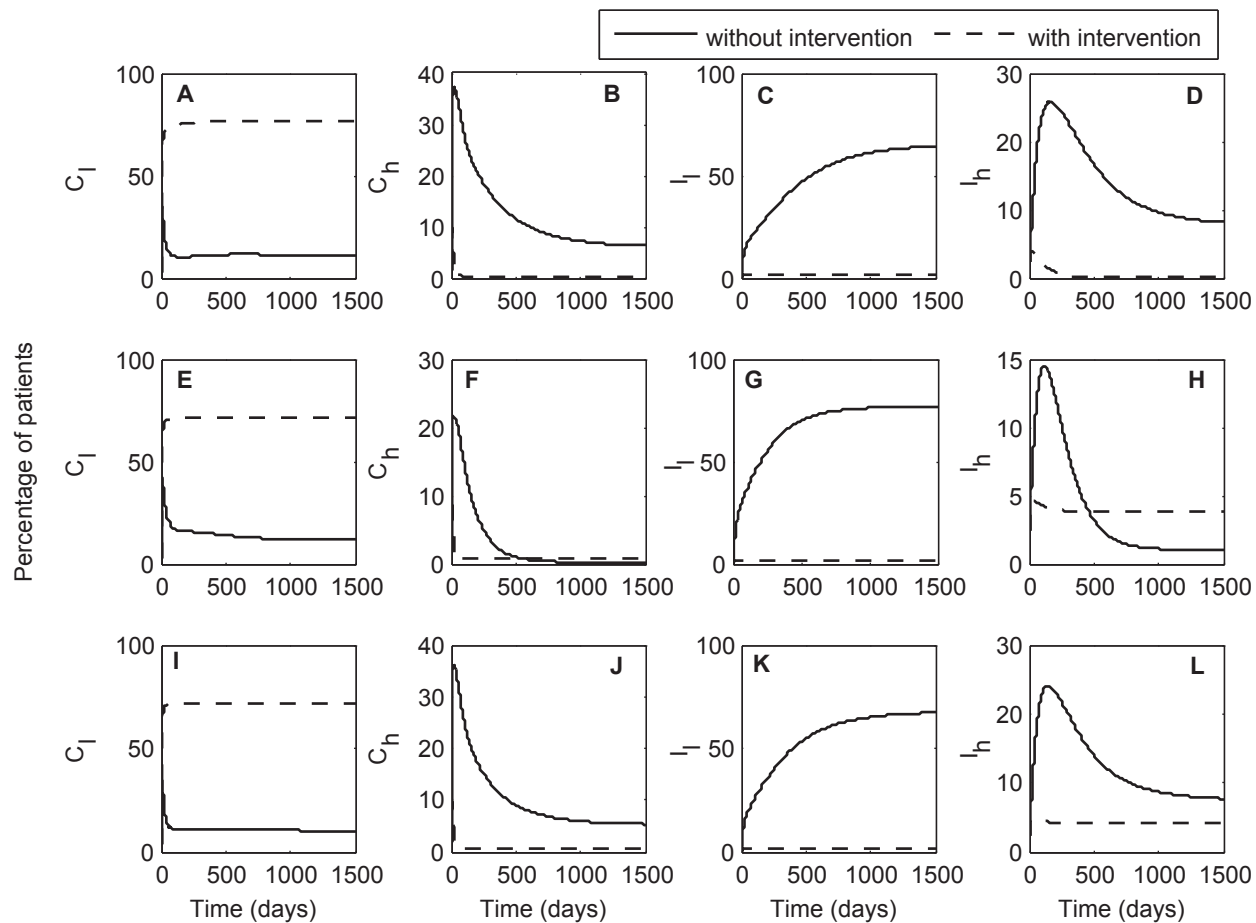

Supplement: Figure S3 — Steady state analyses before and after combined intervention strategies ( µc = 1 and θh = 1) at baseline with admission of colonized patients ( λl = 0.05, λh = 0.007) ( A – D ), with admission of infected ( λil = 0.005, λih = 0.0017) ( E – H ) and with admissions both infected and colonized patients ( λcl = 0.03, λch = 0.07, λil = 0.005, λih = 0.0017) ( I – L ). See Table S1 for other baseline values. (PDF) [file pone.0050688.s003.pdf]

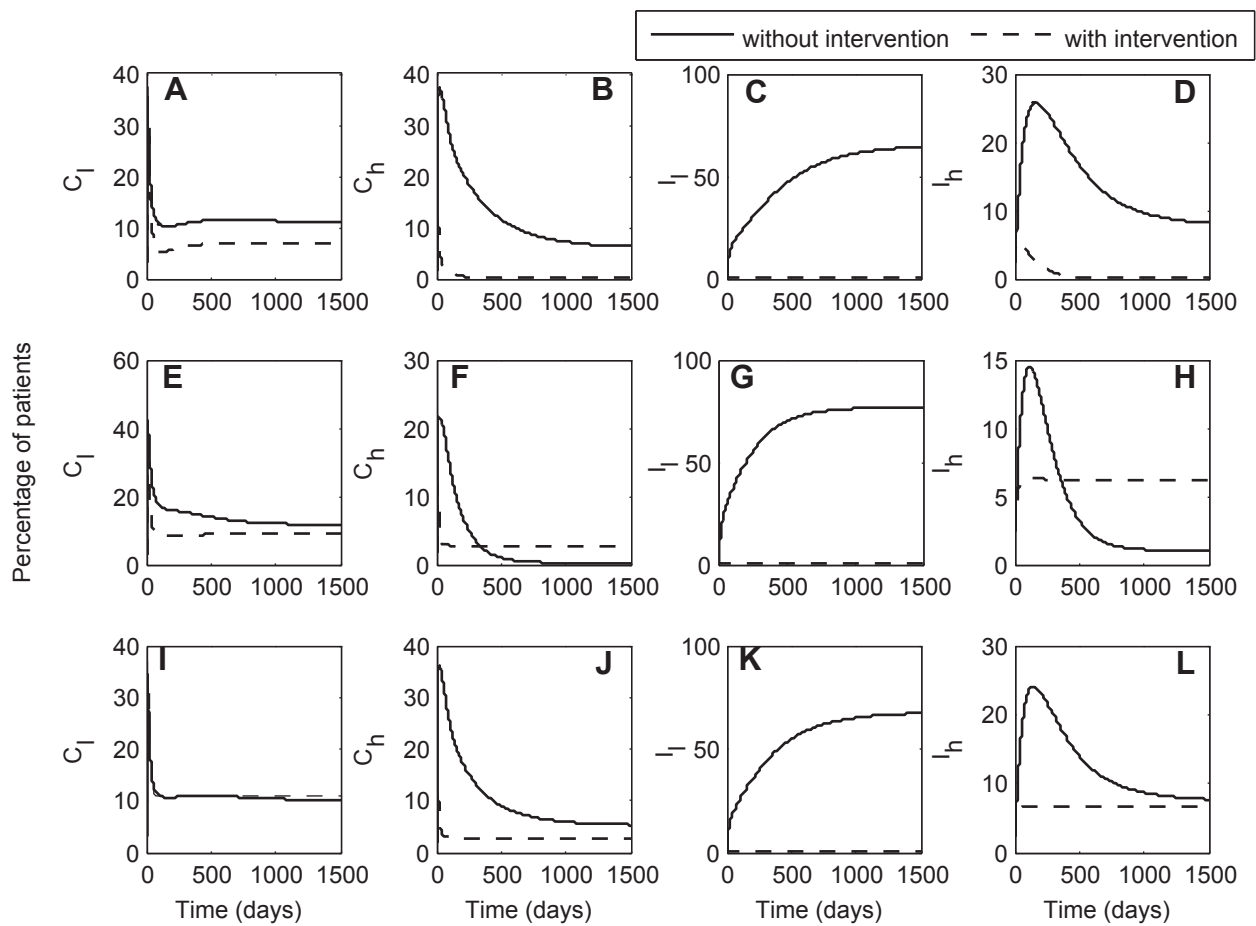

Supplement: Figure S4 — Steady state analyses before and after combined intervention strategies including cross-immunity ( µc = 1, θl = 77.2% and θh = 1) (at baseline with admission of colonized patients ( λl = 0.05, λh = 0.007) ( A – D ), with admission of infected ( λil = 0.005, λih = 0.0017) ( E – H ) and with admissions both infected and colonized patients ( λcl = 0.03, λch = 0.07, λil = 0.005, λih = 0.0017) ( I – L ). See Table S1 for other baseline values. (PDF) [file pone.0050688.s004.pdf]
