# Supplementary material for: Controlling Antimicrobial Resistance through Targeted, Vaccine-Induced Replacement of Strains
Source: PLoS One. 2012 Dec 5;7(12):e50688. doi: 10.1371/journal.pone.0050688 (PMC3515573; doi:10.1371/journal.pone.0050688)
Supplement: Table S1 — Parameter values for the transmission dynamics of Methicillin-Resistant Staphylococcus aureus genotypes, obtained from D’Agata et al. (25), and additional parameters used in this study. (DOCX) [file pone.0050688.s005.docx]

| Variable | Symbol | Baseline value |
| --- | --- | --- |
| Total initial no. of patients | N | 400 |
| Admissions per day | Λ | 70 |
| Admission rate of patients colonized with TTG | λ_cl_ | 0.03 |
| Admission rate of patients colonized with VTG | λ_ch_ | 0.07 |
| Admission rate of patients infected with TTG | λ_il_ | 0.005 |
| Admission rate of patients infected with VTG | λ_ih_ | 0.0017 |
| Colonization rate by colonized TTG patients | β_cl_ | 0.87* |
| Colonization rate by colonized VTG patients | β_ch_ | 0.71* |
| Colonization rate by infected TTG patients | β_il_ | 0.19* |
| Colonization rate by infected VTG patients | β_ih_ | 0.17* |
| Rate of infection with TTG | φ_l_ | 0.02 |
| Rate of infection with VTG | φ_h_ | 0.0143 |
| Discharge rate of susceptible | η_s_ | 0.2 |
| Discharge rate of TTG colonized patients | η_cl_ | 0.2 |
| Discharge rate of VTG colonized patients | η_ch_ | 0.1429 |
| Death rate of TTG infected patients | η_il_ | 0.0033 |
| Death rate of VTG infected patients | η_ih_ | 0.0111 |
| Vaccination efficacy for VTG | θ_h_ | varied |
| Cross-immunity efficacy for TTG | θ_l_ | 0* |
| Treatment rate for TTG | μ_l_ | varied |
| Treatment rate for VTG | μ_h_ | 0 |
| Vaccination coverage | χ | 1 |

*estimates given are baseline in most simulations unless otherwise stated.
