# Supplementary material for: Controlling Antimicrobial Resistance through Targeted, Vaccine-Induced Replacement of Strains
Source: PLoS One. 2012 Dec 5;7(12):e50688. doi: 10.1371/journal.pone.0050688 (PMC3515573; doi:10.1371/journal.pone.0050688)
Supplement: Appendix S1 — Steady State, R0 and vaccine efficacy (EV) analyses. (PDF) [file pone.0050688.s006.pdf]

Steady State Analysis and Calculation of R_o

We present the steady states analyses of the model shown in Table S2. We restrict our analysis to the case with no admission of colonized or infected patients to the hospital, $\lambda_{cl} = \lambda_{ch} = \lambda_{il} = \lambda_{ih} = 0$. By setting the time derivatives in Table S2 to zero and solving the equations, we find a disease-free state

$$E_0 = \left(S_t^0, C_{tl}^0, C_{th}^0, I_{tl}^0, I_{th}^0, S_v^0, C_{vl}^0, C_{vh}^0, I_{vl}^0, I_{vh}^0\right) = \left(\frac{\Lambda}{\eta_s}(1-\chi), 0,0,0,0, \frac{\Lambda\chi}{\eta_s}, 0,0,0,0\right) \qquad \text{(S1)}$$

To study the existence/stability of E_o, we need to compute the basic reproductive number (R_o) associated with E_0. We reorder the components of E_o so that we can separate the two genotypes (TTG-MRSA and VGT-MRSA) easily. The reordered E_0 is

$$\bar{E}_0 = \left(C_{tl}^0, I_{tl}^0, C_{vl}^0, I_{vl}^0, C_{th}^0, I_{th}^0, C_{vh}^0, I_{vh}^0, S_t^0, S_v^0\right) = \left(0,0,0,0,0,0,0,0, \frac{\Lambda}{\eta_s}(1-\chi), \frac{\Lambda\chi}{\eta_s}\right) \qquad \text{(S2)}$$

According to Driessche & Watmough [van den Driessche, P. & Watmough, J. 2002 Reproduction numbers and sub-threshold endemic equilibria for compartmental models of disease transmission. Math. Biosci. 180, 29–48.], R_o is given by the spectral radius (dominant eigenvalue) of the matrix FV^{-1}, where F and V are

$$F = \left[\frac{\partial F_i}{\partial X_j}\right]\Bigg|_{\bar{E}_0} \qquad \text{(S3)} \qquad \text{and} \qquad V = \left[\frac{\partial V_i}{\partial X_j}\right]\Bigg|_{\bar{E}_0} \qquad \text{(S4)}$$

respectively. In our model, X_j denote each component of $\left(C_{tl}, I_{tl}, C_{vl}, I_{vl}, C_{th}, I_{th}, C_{vh}, I_{vh}\right)$; F_i and V_i are

$$\begin{bmatrix} F_1 \\ F_2 \\ F_3 \\ F_4 \\ F_5 \\ F_6 \\ F_7 \\ F_8 \end{bmatrix} = \begin{bmatrix} S_t(\frac{\beta_{cl}}{T}C_{tl} + \frac{\beta_{il}}{T}I_{tl}) \ + \ S_t(1-\theta_l)(\frac{\beta_{cl}}{T}C_{vl} + \frac{\beta_{il}}{T}I_{vl}) \\ 0 \\ S_v(1-\theta_l)(\frac{\beta_{cl}}{T}C_{vl} + \frac{\beta_{il}}{T}I_{vl}) \ + \ S_v(\frac{\beta_{cl}}{T}C_{tl} + \frac{\beta_{il}}{T}I_{tl}) \\ 0 \\ S_t(\frac{\beta_{ch}}{T}C_{th} + \frac{\beta_{ih}}{T}I_{th}) \ + \ S_t(1-\theta_h)(\frac{\beta_{ch}}{T}C_{vh} + \frac{\beta_{ih}}{T}I_{vh}) \\ 0 \\ S_v(1-\theta_h)(\frac{\beta_{ch}}{T}C_{vh} + \frac{\beta_{ih}}{T}I_{vh}) \ + \ S_v(\frac{\beta_{ch}}{T}C_{th} + \frac{\beta_{ih}}{T}I_{th}) \\ 0 \end{bmatrix} \tag{S5}$$

and

$$\begin{bmatrix} V_1 \\ V_2 \\ V_3 \\ V_4 \\ V_5 \\ V_6 \\ V_7 \\ V_8 \end{bmatrix} = \begin{bmatrix} -\mu_l I_{tl} + \alpha_l C_{tl} + \phi_l C_{tl} + \eta_{cl} C_{tl} \\ -\phi_l C_{tl} + \mu_l I_{tl} + \eta_{il} I_{tl} \\ -\mu_l I_{vl} + \alpha_l C_{vl} + \phi_l C_{vl} + \eta_{cl} C_{vl} \\ -\phi_l C_{vl} + \mu_l I_{vl} + \eta_{il} I_{vl} \\ -\mu_h I_{th} + \alpha_h C_{th} + \phi_h C_{th} + \eta_{ch} C_{th} \\ -\phi_h C_{th} + \mu_h I_{th} + \eta_{ih} I_{th} \\ -\mu_h I_{th} + \alpha_h C_{th} + \phi_h C_{th} + \eta_{ch} C_{th} \\ -\phi_h C_{vh} + \mu_h I_{vh} + \eta_{ih} I_{vh} \end{bmatrix} \tag{S6}$$

respectively. Using (S3)-(S6), we can find

$$F=\begin{bmatrix} \frac{\beta_{cl}S_t^0}{T} & \frac{\beta_{il}S_t^0}{T} & \frac{S_t^0(1-\theta_l)\beta_{cl}}{T} & \frac{S_t^0(1-\theta_l)\beta_{il}}{T} & 0 & 0 & 0 & 0 \\ 0 & 0 & 0 & 0 & 0 & 0 & 0 & 0 \\ \frac{\beta_{cl}S_v^0}{T} & \frac{\beta_{il}S_v^0}{T} & \frac{S_v^0(1-\theta_l)\beta_{cl}}{T} & \frac{S_v^0(1-\theta_l)\beta_{il}}{T} & 0 & 0 & 0 & 0 \\ 0 & 0 & 0 & 0 & 0 & 0 & 0 & 0 \\ 0 & 0 & 0 & 0 & \frac{\beta_{ch}S_t^0}{T} & \frac{\beta_{ih}S_t^0}{T} & \frac{S_t^0(1-\theta_h)\beta_{ch}}{T} & \frac{S_t^0(1-\theta_h)\beta_{ih}}{T} \\ 0 & 0 & 0 & 0 & 0 & 0 & 0 & 0 \\ 0 & 0 & 0 & 0 & \frac{\beta_{ch}S_v^0}{T} & \frac{\beta_{ih}S_v^0}{T} & \frac{S_v^0(1-\theta_h)\beta_{ch}}{T} & \frac{S_v^0(1-\theta_h)\beta_{ih}}{T} \\ 0 & 0 & 0 & 0 & 0 & 0 & 0 & 0 \end{bmatrix} \equiv \begin{bmatrix} F_L & O \\ O & F_H \end{bmatrix}$$

(S7)

and

$$V=\begin{bmatrix} \alpha_l+\phi_l+\eta_{cl} & -\mu_l & 0 & 0 & 0 & 0 & 0 & 0 \\ -\phi_l & \mu_l+\eta_{il} & 0 & 0 & 0 & 0 & 0 & 0 \\ 0 & 0 & \alpha_l+\phi_l+\eta_{cl} & -\mu_l & 0 & 0 & 0 & 0 \\ 0 & 0 & -\phi_l & \mu_l+\eta_{il} & 0 & 0 & 0 & 0 \\ 0 & 0 & 0 & 0 & \alpha_h+\phi_h+\eta_{ch} & -\mu_h & 0 & 0 \\ 0 & 0 & 0 & 0 & -\phi_h & \mu_h+\eta_{ih} & 0 & 0 \\ 0 & 0 & 0 & 0 & 0 & 0 & \alpha_h+\phi_h+\eta_{ch} & -\mu_h \\ 0 & 0 & 0 & 0 & 0 & 0 & -\phi_h & \mu_h+\eta_{ih} \end{bmatrix} \equiv \begin{bmatrix} V_L & O \\ O & V_H \end{bmatrix} \quad \text{(S8)}$$

where F_L, F_H, V_L, V_H are 4 by 4 matrices and O is a 4 by 4 matrix of zeros.

The basic reproductive numbers for the TTG-MRSA strain in the absence of VTG-MRSA strain is

$$R_0^L = \rho\left(F_L V_L^{-1}\right) \qquad \text{(S9)}$$

where $\rho\left(A\right)$ denotes the spectral radius (dominant eigenvalue) of the matrix *A*.

Similarly, the basic reproductive number for the VGT-MRSA strain in the absence of TTG-MRSA strain is

$$R_0^H = \rho\left(F_H V_H^{-1}\right) \qquad \text{(S10)}$$

Finally, the basic reproductive number for the whole system is $R_0 = \max\left\{R_0^L, R_0^H\right\}$. If $R_0 < 1$, the steady state E_0 is stable; otherwise E_0 is unstable.

Calculating Efficacy of Vaccine (EV) during the lag period

As mentioned in the text, the lag period for host immunity to build after vaccination against the pathogen in question takes several days (assumed to be *n* days). Due to this fact there will be high incidence of MRSA infection, depending on the force of infection (β_i), before the vaccine fully protects the individuals. To take this fact into account in our model, we explored the efficacy of vaccine (*EV*) during the lag period.

Assume an individual gets vaccinated at time t_0 and the vaccine efficacy after vaccination is determined by a strict increasing function $f(t)$ that satisfies $f(t_0) = 0$ and $f(t_0 + n) = 1$. Without loss of generality, we let $t_0 = 0$ in the rest of this analysis. We defined the *EV* as the probability that the vaccinated individual will not get infected within *n* days after vaccination. Specifically,

$$EV = \prod_{j=1}^{n}\left(1 - P_j\right) \qquad \text{(S11)}$$

where P_j is the probability that the vaccinated individual get infected on *j-th* day after vaccination,

$$
\begin{aligned}
P_1 &= (1 - f(0))\beta_i \\
P_2 &= (1 - f(1))\beta_i \\
P_3 &= (1 - f(2))\beta_i \qquad \text{(S12)} \\
&\vdots \\
P_n &= (1 - f(n-1))\beta_i
\end{aligned}
$$

Note that we have used the vaccine efficacy at the beginning of the j-th day (instead of the efficacy at the end of that day) to estimate P_j conservatively.

Substituting (S12) into (S11) gives

$$
EV = \prod_{j=1}^{n} \left[1 - (1 - f(j))\beta_i \right] \qquad \text{(S13)}
$$

As an example, let $n = 10$, $\beta_i = 0.17$, and $f(t) = \frac{\tanh(t/2)}{\tanh(n/2)}$, we find that $EV = 71\%$ from (S13).
